# Understanding congestion propagation by combining percolation theory with the macroscopic fundamental diagram

**DOI:** 10.1038/s42005-023-01144-w

**Published:** 2023-02-01

**Authors:** Lukas Ambühl, Monica Menendez, Marta C. González

**Affiliations:** 1 Institute for Transport Planning and Systems, ETH Zurich, Zurich, Switzerland; 2 Division of Engineering, New York University Abu Dhabi, Abu Dhabi, United Arab Emirates; 3 Department of City and Regional Planning and Civil and Environmental Engineering, University of California, Berkeley, CA USA

**Keywords:** Statistical physics, thermodynamics and nonlinear dynamics, Applied physics

## Abstract

The science of cities aims to model urban phenomena as aggregate properties that are functions of a system’s variables. Following this line of research, this study seeks to combine two well-known approaches in network and transportation science: (i) The macroscopic fundamental diagram (MFD), which examines the characteristics of urban traffic flow at the network level, including the relationship between flow, density, and speed. (ii) Percolation theory, which investigates the topological and dynamical aspects of complex networks, including traffic networks. Combining these two approaches, we find that the maximum number of congested clusters and the maximum MFD flow occur at the same moment, precluding network percolation (i.e. traffic collapse). These insights describe the transition of the average network flow from the uncongested phase to the congested phase in parallel with the percolation transition from sporadic congested links to a large, congested cluster of links. These results can help to better understand network resilience and the mechanisms behind the propagation of traffic congestion and the resulting traffic collapse.

## Abstract (web summary)

This study analyzes the way car traffic networks collapse by connecting classical traffic flow descriptors with percolation theory: The maximum average car traffic flow in a network and the maximum number of congested clusters occur at the same moment, precluding the network percolation.

## Introduction

Urban traffic networks act as important engines of urban growth and economic prosperity but also place limits on their sustainability, health, and quality of life. Thus, understanding the collapse of traffic networks (i.e. network-wide congestion) influences land use and environmental policies. Current methods of modeling the dynamics of traffic congestion lack cohesion, especially when they come from different fields. Current studies either investigate the macroscopic traffic dynamics^1–5^, or they analyze the spatio-temporal congestion propagation at the network level^6–10^.

**Fig. 1:**
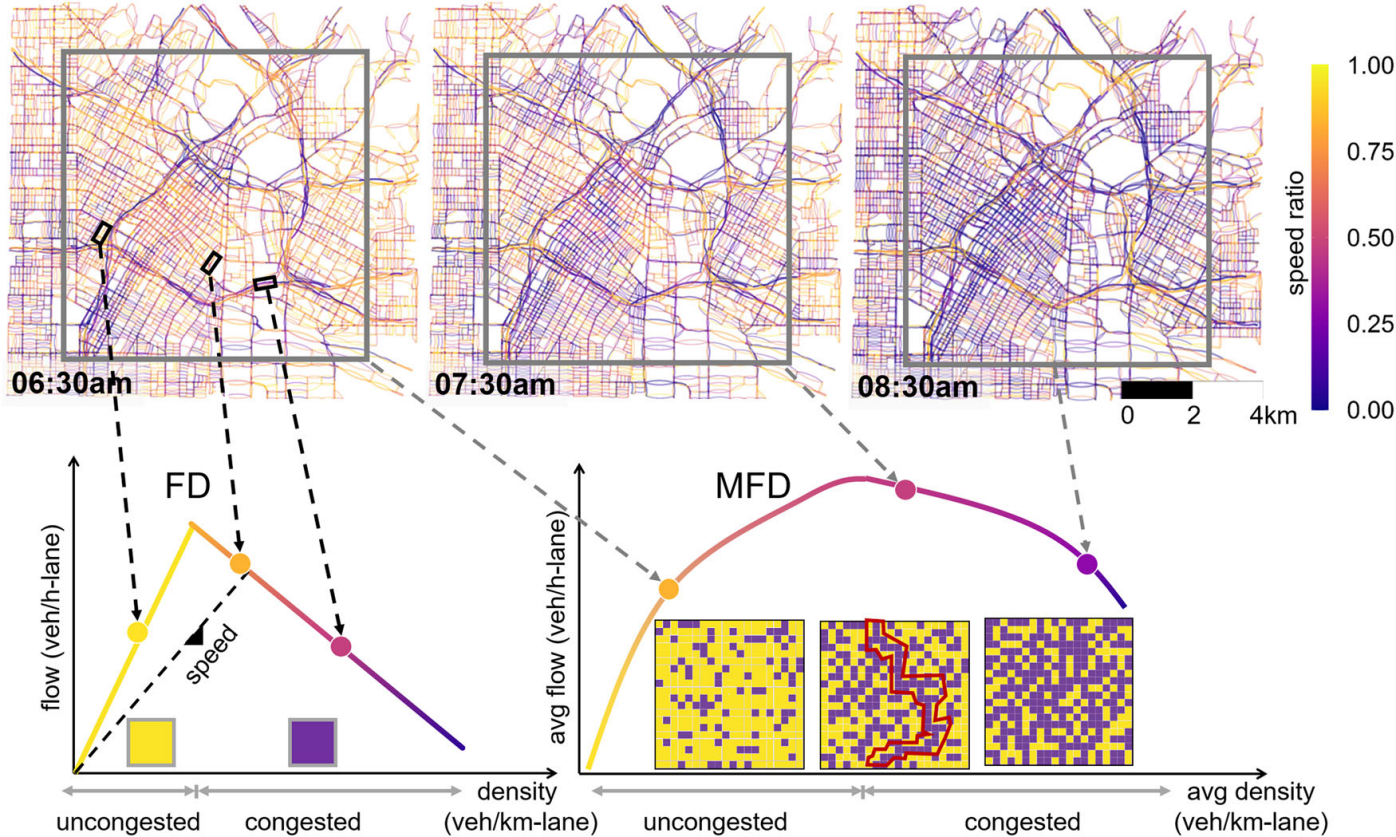
Examples of link and network level perspectives compared. The figure shows traffic conditions (measured in speed ratio) at 06:30, 07:30, and 08:30 in Downtown Los Angeles, respectively. The speed ratios are obtained by dividing traffic speed by the speed limit. Traffic flow on a single link is described by a fundamental diagram (FD). Here, we show a triangular version thereof, where we identify two states: uncongested (increasing slope above the yellow box) and congested (decreasing slope above the purple box). At the network level, the macroscopic fundamental diagram (MFD) describes the traffic dynamics for urban road networks. We link the two perspectives using percolation represented by squares (sites) of an infinite lattice. Each square in the lattice has a certain probability *p* of being congested (purple) and 1−*p* of being uncongested (yellow). This is analogous to a traffic network, where each link is either uncongested or congested as defined by a fundamental diagram. Percolation models the likelihood of finding a path from the top to the bottom of the lattice via congested (purple) sites based on the value of *p*. An example of such a path is marked in red in the lattice in the middle.

Since the beginning of traffic research, a few key descriptors of traffic dynamics on individual links have proven useful. Among the most prominent are the Fundamental Diagram^9,11–13^ and the Bureau of Public Roads (BPR) function^14^. According to the fundamental diagram as shown in Fig. 1, initially uncongested roads will allow traffic flow to increase as vehicle density increases. However, once the density exceeds a certain value, the flow begins to decrease resulting in congestion. Similarly, the BPR function, named after the Bureau of Public Roads, describes traffic congestion on a link as a function of its traffic volume divided by its theoretical capacity. Despite the success of these link level metrics, they neglect the interactions with other roads. As a result, they do not provide information about large scale traffic congestion.

Network level perspectives seek to remedy this by capturing the complexities of traffic dynamics throughout the full road network. Many such indicators have been proposed including travel time^15,16^, observed network flows^17^, and network capacities^18,19^. At their core, these all attempt to extend successful link level metrics in the hopes of accurately describing the full complex network through a single, simple to understand quantity. The MFD represents a network level analogy of the fundamental diagram. It does so by relating the average network flow to the average vehicle density. The MFD is widely used in traffic engineering since it allows, with local data from loop detectors, to parsimoniously model, manage, and optimize traffic in urban networks.

Existing literature has demonstrated the importance of such inter-link communication^1,6,20–25^. Pioneering work in the last few years established that traffic networks exhibit a percolating state based on the characteristic speeds in their roads^6,21^. Follow-up works have confirmed this fact, a recent example of this is the use of a susceptible-infected-recovered (SIR) model to predict^20^ traffic congestion propagation. This is a general approach based on percolation theory^26,27^. Many types of networks exhibit percolation processes where drastic changes in the geometric structure of congested clusters can occur, analogous to traffic congestion spreading as seen in Fig. 1. A simple example of percolation is given by the lattices shown in Fig. 1^28^. Here, the sites (squares) of an infinite lattice have a probability *p* of being congested and 1−*p* of being uncongested. For small *p* (i.e. few congested sites), there exist several small clusters (connected purple squares). Conversely, for large *p*, a single large cluster of the same order as the system develops. Note, the transition between scenarios is very drastic, occurring at what is called the critical point.

Despite the rich literature on both the network science and the traffic engineering side, these two perspectives have not yet been unified into a single Science of Traffic Networks consistently describing the dynamics of the congestion. How is the percolation phenomenon linked to the aggregated traffic states observed in the network and depicted by the MFD? In this work, we address this issue by connecting the two perspectives for five diverse cities worldwide. We begin by modeling the traffic networks through simulations spanning an extended morning traffic peak. These networks incorporate high-resolution features including road classes, priorities, lane geometries, and traffic signals. We combine this framework with calibrated origin-destination matrices and state-of-the-art traffic simulations in order to emulate realistic conditions. We then assess the network level traffic performance and connect multiple network level measures. We uncover a relationship between the MFD and percolation theory, which allows us to arrive at a surprisingly clear connection between the network and link level perspectives: the number of congested clusters of percolation and the average flow in the network (MFD) follow the same function across the day - the correlation coefficient is 0.93. Moreover, the number of congested clusters reaches its maximum simultaneously with the global system flow (in veh/h) represented by the maximum of the MFD. From a percolation perspective, the depicted behavior of the number of congested clusters is a concomitant phenomenon of a percolation process - it is what we call a precursor for percolation. In other words, once the MFD flow starts to decrease, congested clusters start to merge, hence their number decrease, which in turn is the precursor of the very quick formation of a congested cluster spanning a large part of the network - i.e. percolation. Therefore, we argue that the uniting element of the MFD and percolation is the maximum number of clusters. Therefrom we conjecture that the MFD and the percolation process are transitively connected to each other. These results connect the MFD and percolation theory.

Our findings, therefore, provide a clear understanding of the traffic response in cities. This, in turn, can affect their planning of space and demand for mitigating congestion’s negative impacts.

## Results

### Approach

**Fig. 2:**
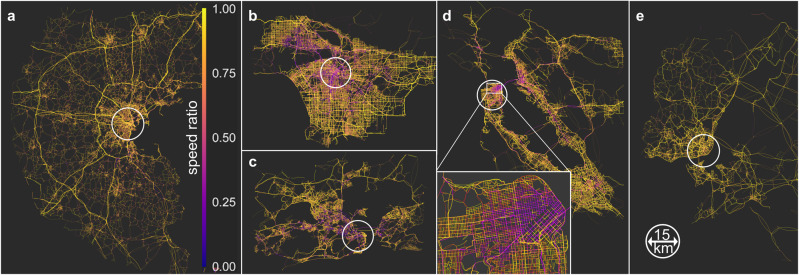
Maps of the traffic networks for each city. The networks depicted represent the morning traffic conditions (at 09:00) on a weekday in **a** Boston, **b** Los Angeles, **c** Rio de Janeiro, **d** San Francisco Bay Area, and **e** Lisbon. The speed ratios are obtained by dividing traffic speed by the speed limit.

We analyze spatiotemporal traffic patterns using state-of-the-art traffic simulations^29^ of five metropolitan areas: Boston, Lisbon, Los Angeles, Rio de Janeiro, and San Francisco. Origin-Destination (OD) tables for traffic demands were initially derived from call detail records (CDR), calibrated, and used in previous studies^30–32^. Figure 2 depicts each of these networks to scale. The Fig. 2d inset magnifies downtown San Francisco highlighting the details of the simulation. Unlike other models, the extent and detail of the network allow for realistic congestion spreading at high spatiotemporal resolution. Our analysis focuses on an extended morning peak hour (05:30-11:00). Details on the networks, simulation framework, and travel time validation are given in Methods and Supplementary Note 2.

### Network level traffic performance

In the past decade, the traffic engineering community has employed a conservation-based reservoir modeling of city-wide traffic congestion^3,4,18^. It is best illustrated by a bathtub where the water inflow refers to cars joining the traffic stream, water draining from the bathtub to cars leaving it by exiting the network or ending their trip, and the depth of water in the bathtub to the density of traffic. Such behavior is summarized by the MFD as introduced in Fig. 1. The product of traffic density and speed defines the system’s trip arrivals at destinations per time step. Above some peak flow density, traffic flow (which is proportional to the vehicle-distance traveled) decreases resulting in a lower trip ending rate^2^.

**Fig. 3:**
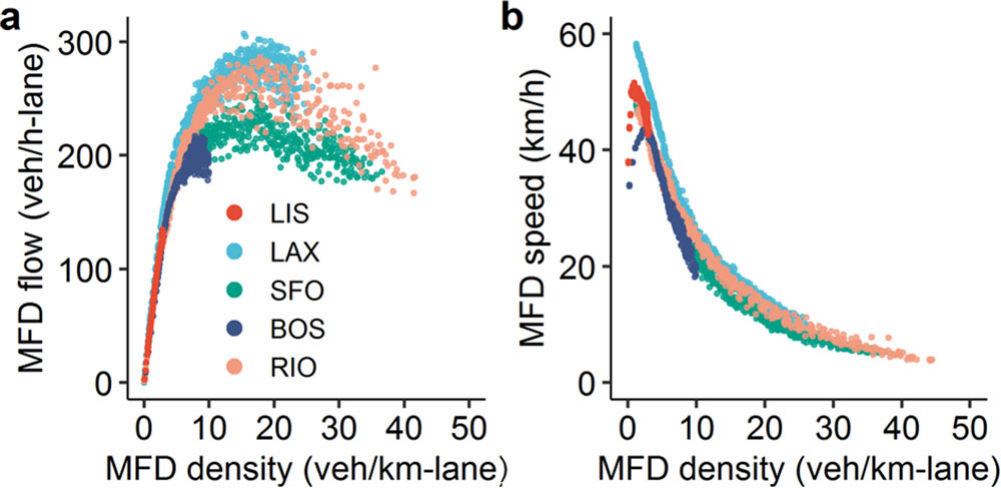
An analysis of the average traffic performance of the central business district of each city. **a** Depicts the measured macroscopic fundamental diagram (MFD) flow versus vehicle density of each city. Note that Lisbon and Boston do not reach the congested branch of the MFD. **b** Shows the MFD speed as a function of vehicle density. Labeling colors are identical to Figure **a**.

Figure 3a, b show the MFDs for the morning peak on the central business district of the five cities analyzed. Each dot represents the average traffic conditions sampled at 5-minutes intervals. Functionally, the MFD flow is defined as:

1 $${{{{{{{\rm{MFD}}}}}}}} \sim X\equiv \frac{\mathop{P}\limits_{l\in}{\ell }_{l}{x}_{l}}{\mathop{P}\limits_{l\in L}{\ell }_{l}}$$

where *X* is the average flow (veh/h-lane; measured over 5-minutes intervals) through the network, *ℓ*_*l*_ is the lane-length (summed over all lanes) of link *l*, *x*_*l*_ is the number of cars that flow through *l* in a given time interval, and *L* is the set of all links. It is important to note that as traffic increases, the average speed of a driver in the network decreases, even before the peak average flow is observed (i.e. while the system is theoretically uncongested). This is evident by the curved top of the MFD, see Fig. 1. Three cities exhibit a decreasing, congested branch in the MFD: San Francisco (SFO), Rio de Janeiro (RIO), and Los Angeles (LAX). Boston (BOS) and Lisbon (LIS) do show decreasing flows in the uncongested branch, but they are the result of a decrease in traffic demand and not traffic congestion.

### Traffic percolation

Following our introductory Fig. 1, we analyze traffic in the five urban networks as a percolation phenomenon. For every time interval, we classify every link as either congested or uncongested using its current traffic condition based on the fundamental diagram (a link level metric). This binary definition is consistent with the traffic dynamics of a road segment. Essentially, it follows the idea of a triangular fundamental diagram. Using a labeling approach that is based on the critical density of the fundamental diagram is a precise and model-independent choice, which is consistent at the link and network level - unlike the speed-dependent definition used by previous studies in traffic percolation (e.g. ref. ^21^). A congestion definition based on speed is ultimately subjective and somewhat arbitrary. This seemingly minor difference enables us to use consistent definitions across both the link and network level perspectives and therefore ultimately unify our results.

To the best of our knowledge, previous studies focusing on percolation did look into how the percolation threshold changed over time depending on the time of the day. They did not, however, look at how the clusters themselves evolved over the time of the day. Here, we track the evolution of congested clusters over time, which has been neglected in previous traffic percolation analyses. We find that while the number and the size of the congested clusters are relatively low in the early morning, both increase with time until the clusters start merging into one large cluster.

**Fig. 4:**
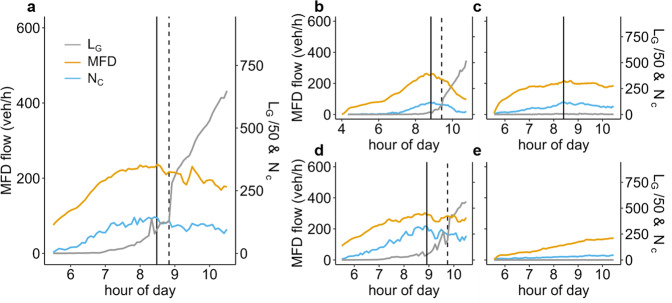
Comparison of the macroscopic fundamental diagram (MFD) and traffic congestion spreading. This figure depicts the temporal evolution of the size of the largest cluster (*L*_*G*_, gray), the MFD flow (veh/h-lane, yellow), and the number of congested clusters (*N*_*c*_, blue) for a selected random seed in **a** San Francisco, **b** Rio de Janeiro, **c** Boston, **d** Los Angeles, and **e** Lisbon. The solid vertical line indicates the time at which the maximum number of clusters emerge, which coincides with the time at which the MFD reaches its maximum flow; the dashed line indicates when the system percolates.

Figure 4 shows the average flow based on the MFD (a network level measure), the number of clusters, and the size of the largest cluster as a function of time. We see that, in San Francisco, Rio de Janeiro, and Los Angeles, the peak network flow occurs when the number of congested clusters reaches its maximum, right before the giant component emerges^28^. While Boston and Lisbon do not reach their maximum possible flow, we still observe a correlation between their average flows and the number of clusters. To the best of our knowledge the average flow in a network has not been linked previously to the spatial propagation of congestion. Traffic throughput depends on the number of links congested and especially on the number of congested clusters. Thus, the MFD, which represents the traffic flow, is evidently linked to the congestion propagation in urban traffic networks. From a statistical perspective, we tested the correlation between the MFD flow and the number of congested clusters and they average 0.93 over all five cities and simulations, hence, confirming the high similarity between the trends from a technical perspective.

Right after reaching the maximum number of clusters, we see a sharp increase in the largest cluster’s size, the giant component (*L*_*G*_), for the cities exhibiting a congested MFD branch. The gray curve represents the evolution of *L*_*G*_. In the beginning, clusters of congestion are locally isolated. With time, they grow in number. Once the clusters start to merge and their overall number drops, these pockets of congestion stop being a local phenomenon. It becomes more difficult (but not impossible) to detour since many links are now congested. The detours get longer and slower; hence, the MFD flow reduces. The clusters remain mostly separated in space until a critical point is reached where the clusters suddenly merge into one giant component. This marks the time instant at which the network percolates. It is common to define the percolation point as the moment at which the second-largest cluster reaches its maximum size^21^. Intuitively, this happens right before it merges with the largest cluster. This moment in time is shown as a vertical dashed line in Fig. 4. From our analysis, it becomes clear that not all cities exhibit percolation under realistic traffic conditions. Congested clusters in Boston and Lisbon do not reach the point of percolation with current traffic demand levels. Still, these cities confirm the high correlation between the number of congested clusters and average MFD-flow, also for the non-percolating networks. Artificially increasing the traffic demand for these cities proportionally would cause their networks’ MFDs to become congested and thereafter percolate like the other cities shown. In this study, we refrain from doing so in order to demonstrate our findings on real networks with real demand levels. A detailed analysis of the cluster size distribution before, at, and after percolation can be found in Supplementary Note 4.

We see that as the MFD reaches its maximum, network congestion spills back across the network, causing congested clusters to merge and eventually bringing about the percolation transition.

**Fig. 5:**
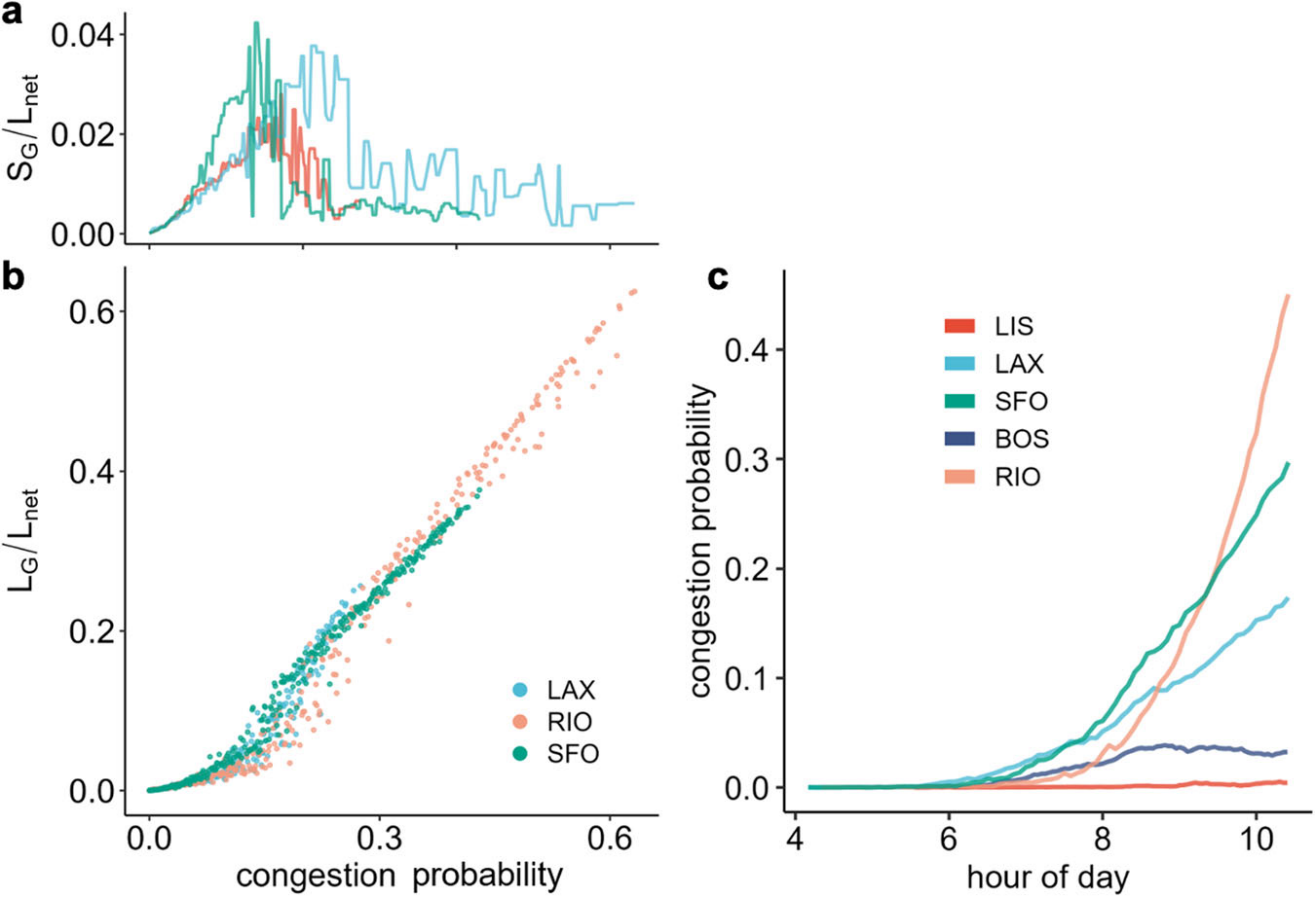
Percolation and congestion probability. **a**, **b** shows the traditional percolation approach as the fraction of links of our network that resides in *S*_*G*_ and *L*_*G*_ vs the congestion probability (includes all 15 random seeds; *S*_*G*_ represents the second largest component, here smoothed across all seeds; *L*_*net*_ represents the size of the network; a shares same colors as **b**). **c** shows the congestion probability over time.

While this helps to unify the different perspectives to understand traffic, the classical implementation of percolation assesses how the system changes with probability rather than time. Figure 5b shows the fraction of links of our network that resides in *L*_*G*_ vs the congestion probability (fraction of congested links). At low congestion probability, *L*_*G*_ contains only a small fraction of the network. Then after the critical point, *L*_*G*_ jumps to a large fraction. Simultaneously, the second-largest component (*S*_*G*_) suddenly drops in value (Fig. 5a). We should note that the critical point appears to shift depending on the city. This may indicate that the effective dimension of the various cities is different because the critical point depends on network characteristics^19,28^. Furthermore, it has been shown recently that the dynamics of traffic flow on city roads and highways result in different effective dimensions^33^. Thus, the variations of the critical point are likely a result of different network compositions (e.g. a change in the ratio of city roads to highways).

In order to better understand how the system changes with the congestion probability, Fig. 5c shows the evolution of the congestion probability over time. Note that the congestion probability is a function of the evolution of the origin-destination matrix over time combined with the network structure. Not surprisingly, the probability increases substantially for the three cities reaching a congested branch of the MFD. Still, the pattern varies significantly, which further underlines the value of our study which unifies congestion propagation under the umbrella of the MFD and percolation.

## Discussion

A century passed the age of the automobile, traffic networks are still being described as engines of global growth and prosperity. However, their uninformed expansion is a leading cause of pollution and other negative externalities. The collapse of traffic networks, i.e. network-wide congestion, causes a loss in social and economic opportunities and increased carbon emissions. In our endeavor towards greener and more livable cities, a clear understanding of traffic network congestion is necessary to simplify science-informed policy.

In this work, we use realistic high-resolution simulations to explore the impact of traffic congestion on urban road networks. We uncover a strong correlation between the percolation of traffic congestion (long-established by network scientists) and the average flow through the network as measured by the MFD (widely used by traffic engineers). Both are connected by the number of congested clusters and the average network flow, for which both reach their maximum simultaneously, serving as a pre-cursor for a percolation of congestion. This surprisingly clear connection between the network and link level perspectives allows us to coherently describe the dynamics of the congestion at both levels simultaneously. Therefore, because percolation is a tool for assessing network resilience, our results have the potential to enable planners to take advantage of the minimal data requirements of the network level measures to assess road network resilience using a single actionable framework.

While the theories and concepts used here have been studied on their own, we take it one step further and show that they are indeed connected. When combined they lay the groundwork for a unification of perspectives, approaches, and fields of study into a single Science of Traffic that updates our understanding of urban traffic substantially. It allows traffic scientists to draw on knowledge from a coherent set of models and approaches and to communicate their results more efficiently to other researchers in the field.

Our congestion definition allows for consistency, but it also might classify roads as congested as per the triangular fundamental diagram, when in fact they still show a relatively high flow, close to capacity. Intuitively, it takes some time for congestion to move upstream connecting multiple saturated roads to create a large congested cluster. This might explain some of the delay between the MFD-flow maximum and the critical point of percolation. Nonetheless, a further sensitivity analysis is necessary in the future to fully describe the magnitude and mechanisms behind such delay. Moreover, our binary definition of congestion based on traffic flow theory does not easily translate to previously used definitions in the field of traffic percolation. Future work could try to relate metrics previously used in traffic percolation, such as the critical speed threshold used in ref. ^21^. Additionally, while we found that our focus on congested clusters offers a better understanding of the spatio-temporal evolution of congestion, an interesting line of future research consists of analyzing uncongested clusters and any relation they might have to the percolation process.

From a traffic perspective, newer and more dynamic approaches in the field allow for a time-dependent definition of an MFD-controlled perimeter. Instead of relatively complicated partitioning algorithms for homogeneous regions, future research could test new frameworks which mostly focus on preventing the merging of two separated clusters (the percolation moment). Instead of controlling somewhat homogeneous regions, it could be interesting to show whether it is possible to apply a similar framework as in ref. ^21^, identify critical bottlenecks in the network, and thereby define new partitioning methodologies and traffic control schemes. Note, such ideas were very recently followed up by ref. ^34^.

Open work in this subject contains, but is not limited to, a further comparison of cities of various sizes, demographics, and network topologies to systematically account for socioeconomic, emissions, and network variance. Our study offers the starting point for future research to understand which relationships exist between traffic demand, network topology, management strategy, and existence and time of percolation. The Science of Traffic Networks will become increasingly important when planning urban road networks to account for the recent urbanization trends and the development of more liveable and greener cities.

## Methods

### Data

The networks were extracted from OpenStreetMap (OSM). When available, the number of lanes, speed limit, and traffic signal locations were extracted directly from OSM databases. In all other cases, heuristics were used to infer the missing information. The networks include all road types – even traffic-relevant residential roads. Origin-Destination (OD) tables for traffic demands were initially derived from call detail records (CDR), calibrated, and used in previous studies^30–32^. The spatial resolution of the origin-destination table corresponds to traffic assignment zones, which, depending on population density, encompass roughly 1-2 km^2^. We discuss the details in Supplementary Note 1.

### Simulation

The simulations are generated with SUMO^29^ using a mesoscopic multi-lane model^35^. Roads are segmented, and traffic dynamics are modeled from segment to segment obeying some capacity and space constraints. This allows for realistic modeling of vehicle queues. Intersections are modeled according to a detailed right of way scheme. We implement a periodic, stochastic routing of vehicles based on the current traffic conditions in the network^29^. Routing is done via a stochastic shortest path method. 50% of the vehicles adhere to their initial route and 50% are re-routed every 6 min. This is similar to common simulation setups^36^. We use a time resolution of 1s and run 15 different random seeds. The details of the models are discussed in Supplementary Note 2.

### Congested links

We use the fundamental diagram of traffic (FD) to find the peak flow density for every link. If the link’s traffic density *k*_*i*_ exceeds the peak flow density ${k}_{i}^{c}$ of its fundamental diagram, we classify it as congested (${k}_{i}(t) \, > \,{k}_{i}^{c}$). See Supplementary Note 3 for more information.

### Supplementary information

Supplementary Information

## Data Availability

The simulation data that support the findings of this study are available in ETH Zurich’s Research Repository with the identifier 10.3929/ethz-b-000584669.
