# Supplementary material for: Understanding congestion propagation by combining percolation theory with the macroscopic fundamental diagram
Source: Commun Phys. 2023 Feb 1;6(1):26. doi: 10.1038/s42005-023-01144-w (PMC11041767; doi:10.1038/s42005-023-01144-w)
Supplement: Supplementary file 1 — Supplementary Information [file 42005_2023_1144_MOESM1_ESM.pdf]

Supplemental Information: Understanding congestion propagation by combining percolation theory with the macroscopic fundamental diagram

Lukas Ambühl[1], Monica Menendez[2] & Marta C. González[3]

[1] *Institute for Transport Planning and Systems, ETH Zurich, Switzerland*

[2] *Division of Engineering, New York University Abu Dhabi, United Arab Emirates*

[3] *Departments of City and Regional Planning and Civil and Environmental Engineering, University of California, Berkeley*

Supplementary Note 1: Data input

Hourly Origin-Destination (OD) tables for traffic demands were derived from call detail records (CDR), calibrated, and used in previous studies [1–4]. The spatial resolution of the ODs corresponds to traffic assignment zones, which depending on population density, encompass roughly 1-2 km^2. Supplemental Fig. 1 shows the number of vehicle trips demanded (per hour) as well as the resulting trip distance distributions for the simulation duration (05:30-11:00). While all trip distances essentially follow a log-normal distribution, the loading patterns differ substantially between the different cities - especially with respect to the peak hour and the inflow rate.

The road networks were extracted from OpenStreetMap (OSM). When available, the number of lanes, speed limit, and traffic signal locations were extracted directly from OSM databases. In all

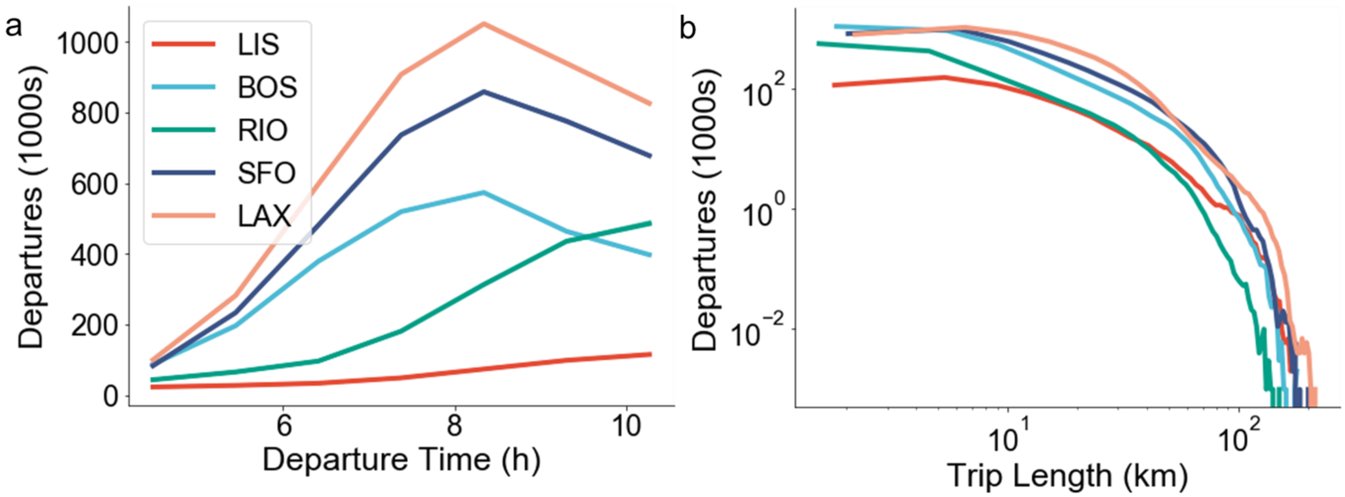

Supplementary Figure 1: (**a**) Number of cars departing from their origin in a given hour versus departure hour. (**b**) Number of cars with a given route length. Note that this is plotted on a log-log scale for ease of reading.

other cases, heuristics were used to infer the missing information[5]. Depending on the city's control policies, the number of lanes, and priority of the incoming links, we implemented heuristics that model intersections either as a traffic signal, a traffic signal allowing right turns on red, an all-way stop, a right-before-left intersection, a full priority intersection, or a zipper merge. Details on the networks are given in Supplementary Table 1. Our networks include all freeways, trunk, primary, secondary, and tertiary roads, as well as a portion of all residential roads. For the latter, we keep only the top 30% of residential roads. We select these by ranking their modified betweenness centrality, where we only consider the shortest paths that depart and arrive on non-residential roads. This way, we ensure that a high betweenness centrality is associated with a residential road that connects roads of higher hierarchy. This process selects roads that are potentially relevant for traffic at the network level. This allows us to substantially decrease the number of possible

routes, and exclude topologically irrelevant residential roads. In addition, the networks are tested and filtered for weak connectivity.

Supplementary Note 2: Simulation

The simulation is performed with “Simulation of Urban Mobility” (SUMO)[5] using a mesoscopic multi-lane model[6], which essentially implements a state-dependent queueing model. Roads are split into 50 m-segments and the traffic dynamics are modeled from segment to segment obeying capacity and spatial constraints. This allows for realistic modeling of vehicle queues. The constraints are defined by a set of vehicle headways. These depend on the segments’ traffic conditions (uncongested/congested) and are responsible for the backward moving waves of traffic congestion. The model, therefore, allows us to describe individual vehicle movements. The traffic conditions on each 50 m-segment of road determine its current allowable flow. The desired driving speed follows a normal distribution around the speed limit with a standard deviation of 0.3. This mimics recent empirical findings[7]. Overtaking is permitted and modeled as a randomized process depending on vehicle speeds and density.

Traffic signal timings are defined separately for every intersection. For a default 4-leg intersection, the cycle time is 90s and the green time is 37s, including a protected phase of 6s for left turns. These values are heuristically adapted for more complicated intersections. Additionally, the mesoscopic model allows scaling the delays at the intersections to take an average level of signal coordination into account, which is sufficient for the simulation at large scale[8]. Intersections are

Supplementary Table 1: Overview of simulation networks.

	Network length [lane-km]	Area [km^2]	Traffic lights	CBD length [lane-km]	Peak Time [hh:mm]	Peak Departures [veh / 5 min]	Total Cars [veh]	Capacity [veh / hr]
BOS	43,756	10,686	4,913	2,816	8:12	52,908	2,615,132	$2.8 \cdot 10^9$
LAX	78,760	12,864	13,667	4,417	8:12	95,821	4,700,531	$7.8 \cdot 10^9$
LIS	32,530	13,675	1,199	1,924	10:39	10,302	419,127	$2.0 \cdot 10^9$
RIO	29,940	6,516	2,774	1,755	10:11	43,540	1,620,927	$2.3 \cdot 10^9$
SFO	56,340	15,806	8,215	2,354	8:12	78,396	3,846,649	$5.0 \cdot 10^9$

modeled according to a detailed right of way scheme, including all-way stops, right turns on red or right-before-left priorities.

For the vehicle routing, we revert to a periodic, stochastic routing of vehicles based on the current traffic conditions in the network[5]. When inserted, vehicles are assigned to a stochastic shortest path, where the speeds on edges are overestimated by a random (uniformly distributed) factor between 1 and 1.7. This mimics recent empirical findings[9].

In summary, our simulations produce results that are consistent with the kinematic wave theory of traffic, while respecting details in the network topology and being computationally reasonably fast[1]. Unlike other models, this combination allows for very detailed and realistic congestion spreading at a high spatial resolution. This is confirmed by the travel time validation in Supplemental Fig. 2.

Supplemental Fig. 2 compares the simulated travel times and the estimated travel times for drivers departing at the same time. The estimates are extracted from Google's Direction API. We randomly analyzed 5,000 trips per city with at least 10 vehicles per hour from our OD-relations. Unlike other simulation validation methods, where travel times are analyzed at the level of traffic assignment zones, we use the travel times of completed trips along with their start and end coordinates as the validation inputs. The plots are shown in a log-log scale, to better illustrate the heavy-tailed travel time distribution. The solid line has a slope of 1 and thus represents equal travel times. Given the scale of the networks and the number of vehicles simulated, we consider the fits

[1]The large-scale simulations are available to academia via the authors' GitHub.

in Supplemental Fig. 2 as of sufficient quality. We attribute the deviations to inconsistencies in the OSM-derived networks, the demand's spatial and temporal resolution, potentially biased travel times from Google[2], a lack of other modes in the simulations, and unknown specifics of the traffic signal operations. This methodology allows recreating a network that behaves realistically.

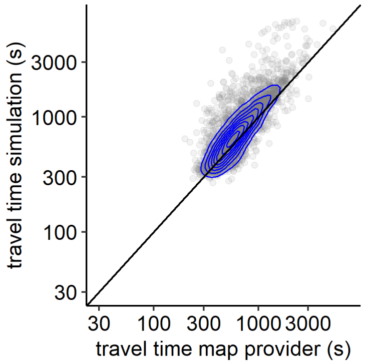

(a) Boston.

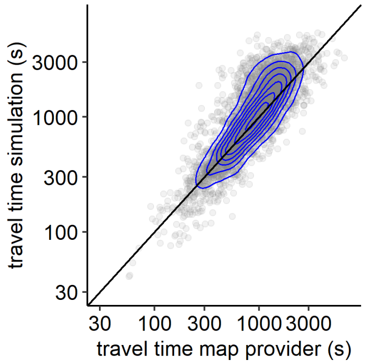

(b) Los Angeles.

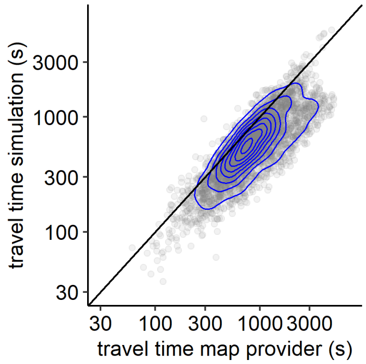

(c) Lisbon.

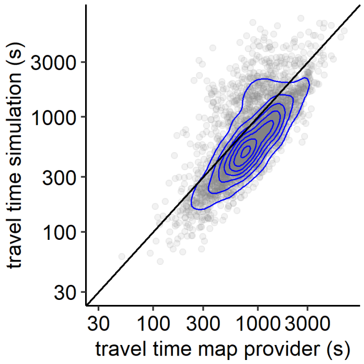

(d) Rio de Janeiro.

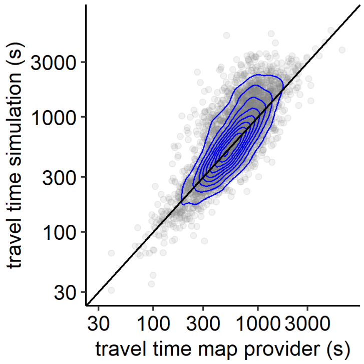

(e) San Francisco.

Supplementary Figure 2: Validation of travel times at the trip level in a log-log plot. Each dot represents the travel time in the simulation vs. the travel time retrieved from Google's Direction API. The contours serve as reading aid.

[2] Google's Direction API does not provide actual travel times, but a smoothed prediction thereof.

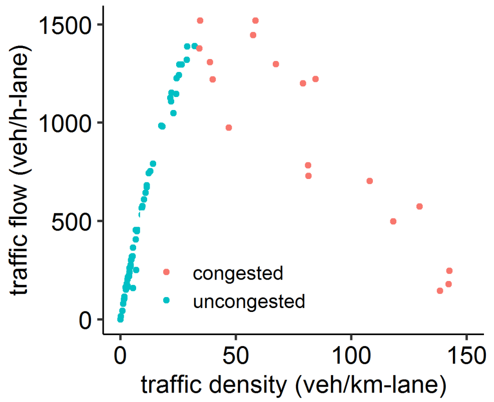

Supplementary Figure 3: Congestion analysis for the Auto Estrada Engenheiro Fernando Mac Dowell in Rio de Janeiro. Each dot represents the 5 min-average traffic conditions as given by the traffic density and flow. We identify points with a traffic density above the peak flow density (which maximizes the flow), as congested and below as uncongested.

Supplementary Note 3: Definition of congestion

We utilize the simulation data to define the fundamental diagram (FD) for every link in the network. We use the FD to determine the peak flow density for every link. We then evaluate for every time interval, whether the link's traffic density k_i exceeds the link's peak flow density k_i^c. This allows us to classify it as congested ($k_i(t) > k_i^c$) or uncongested ($k_i(t) \leq k_i^c$). Figure 3 shows an example of such classification.

Supplementary Note 4: Distribution of cluster size before, at, and after percolation

One property of percolating systems is their special scaling behavior at the point of percolation (criticality). At criticality, the cluster size distribution is scale-free, i.e. follows a power law.

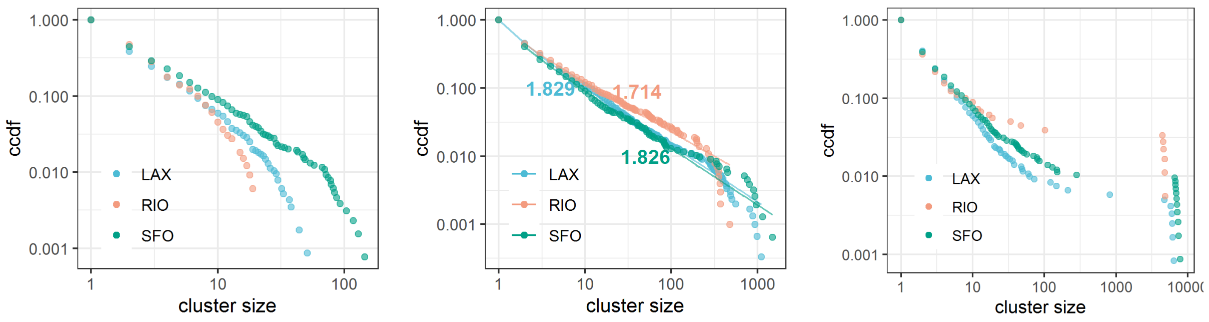

(a) Below critical cluster size ccdf. (b) Critical point cluster size ccdf. (c) Above critical cluster size ccdf.

Supplementary Figure 4: Cluster size distribution before, at, and after criticality. The plots show the complementary cumulative distribution (ccdf),i.e., how probable it is to find congested clusters that are larger than a certain size. Percolation processes are known for their power-law relationship at criticality.

Below and above criticality, however, this is not the case.

The cluster size distribution at criticality n_s is given by $n_s(p_c) \propto s^{-\tau_p}$ with s being the overall cluster size distribution and τ_p the parameter of the power function. Conceptually this means that we have a few clusters covering vast portions of the networks and many smaller ones co-existing. Supplemental Fig. 4 shows the scaling properties below (1.5 h before), at, and above (1.5 h after) criticality. This relationship becomes more distinct and statistically robust when multiple runs of simulations for each city are evaluated jointly. Thus, for the three networks exhibiting percolation phenomena, we pooled their six random seeds. Note that we plotted the complementary cumulative distribution (ccdf), which shows how probable it is to find congested clusters larger than a certain size, on a log-log plot. As expected, we observe a linear relationship, with slope τ_p, at criticality.

At criticality, the τ_p values are statistically significant with p-values between 0.29 and 0.68 (H_0: data is generated from a power-law distribution). As expected, the p-values for the distributions at non-critical instances in time are below a 0.1 cut-off[10].

We see that τ_p is stable across the cities. This indicates that the three cities essentially follow the same percolation model. This is remarkable considering the substantial differences in the networks, demands, congestion evolution, and even intersection queuing.

Supplementary References

1. Çolak, S., Lima, A. & González, M. C. Understanding congested travel in urban areas. *Nature Communications* **7**, 10793 (2016).

2. Çolak, S., Alexander, L. P., Alvim, B. G., Mehndiratta, S. R. & González, M. C. Analyzing Cell Phone Location Data for Urban Travel. *Transportation Research Record* **2526**, 126–135 (2016).

3. Jiang, S. *et al.* The TimeGeo modeling framework for urban mobility without travel surveys. *Proceedings of the National Academy of Sciences* **113**, 5370–5378 (2016).

4. Xu, Y. & González, M. C. Collective benefits in traffic during mega events via the use of information technologies. *Journal of The Royal Society Interface* **14** (2017).

5. Lopez, P. A. *et al.* Microscopic Traffic Simulation using SUMO. In *IEEE Conference on Intelligent Transportation Systems, ITSC*, 2575–2582 (IEEE, 2018).

6. Eissfeldt, N. G. *Vehicle-based modelling of traffic. Theory and application to environmental impact modelling*. Ph.D. thesis, Universität zu Köln (2004).

7. Li, M., Yu, L., Zhai, Z., He, W. & Song, G. Development of emission factors for an urban road network based on speed distributions. *Journal of Transportation Engineering* **142**, 04016036 (2016).

8. Girault, J.-T., Gayah, V. V., Guler, I. & Menendez, M. Exploratory Analysis of Signal Coordination Impacts on Macroscopic Fundamental Diagram. *Transportation Research Record: Journal of the Transportation Research Board* **2560**, 36–46 (2016).

9. Zhu, S. & Levinson, D. Do people use the shortest path? An empirical test of wardrop's first principle. *PLoS ONE* **10**, e0134322 (2015).

10. Zhang, L. *et al.* Scale-free resilience of real traffic jams. *Proceedings of the National Academy of Sciences* **116**, 8673–8678 (2019).
